# Grape seed proanthocyanidin extract inhibits DNA and protein damage and labile iron, enzyme, and cancer cell activities

**DOI:** 10.1038/s41598-022-16608-2

**Published:** 2022-07-20

**Authors:** Hosam M. Habib, Esmail M. El-Fakharany, E. Kheadr, Wissam H. Ibrahim

**Affiliations:** 1 Functional Foods and Nutraceuticals Laboratory (FFNL), Dairy Science and Technology Department, Faculty of Agriculture, Alexandria University, Alexandria, Egypt; 2 Protein Research Department, Genetic Engineering and Biotechnology Research Institute GEBRI, City for Scientific Research and Technology Applications (SRTA City), New Borg El Arab, Alexandria 21934 Egypt; 3 Department of Nutrition and Health, College of Medicine and Health Sciences, United Arab Emirates University, PO Box 15551, Al Ain, UAE

**Keywords:** DNA, Enzymes, Cancer, Pharmaceutics, Toxicology

## Abstract

Grape seed extract from (*Vitis vinifera*) (VGSE) is an excellent source of various polyphenols that exhibit highly potent antioxidant and disease prevention properties. Although numerous biological activities, with potential for improving human health, have been reported for VGSE, there is a lack of data relating to the health benefits of VGSE on DNA damage, protein damage, labile iron activity, and enzyme inhibitory effects. This investigation demonstrated, for the first time, that VGSE inhibits DNA and BSA damage and labile iron activity in-vitro. Moreover, VGSE also inhibited in-vitro activities of AChE, tyrosinase, and α-amylase. VGSE treatment significantly reduced viability of MCF-7, Hep-G2, Caco-2, and Huh-7 cells after 48-h treatments. The results obtained provide additional support for the purported health benefits of VGSE and reinforce its potential in disease prevention and therapy, especially in relation to cancer.

## Introduction

Grape (*Vitis vinifera*) is one of the major fruit crops produced worldwide (FAO Statistical Data Base, n.d.)^1^, and it has been heralded as a folk medicinal plant with crucial roles in health care and improving multiple human disorders. Grapes can grow all over the world and considered to be the most widely planted fruit crop and the most consumed fruit worldwide^2^. Grape seeds, which are by-products of juice and wine production, are a rich source of biologically active compounds owing to their high concentration of polyphenols^3^. Approximately 60–70% of grape polyphenols are found in the seeds. It is also a greater source of antioxidants than the by-products of grape juice^3^. The seeds account for approximately 5% of the weight of the whole grape, representing approximately 40–50% of solid waste that different wine industries generated during winemaking processes^4^.

Grape seed extract from (*Vitis vinifera*) (VGSE) is considered a major source of anti-toxic substances such as flavonoids, gallic acids, catechin, epicatechin, and proanthocyanidin. It also contains approximately 35% fiber with 29% extractable components including phenolic compounds, proteins (11%), minerals (3%), and water (7%)^5^. VGSE polyphenols have been shown to possess potent anti-inflammatory, hepatoprotective, cardioprotective, anti-mutagenic, and anti-diabetic properties^6^. Moreover, VGSE has shown promising anticancer effects in various cancers^7,8^ through strong prophylactic effects against free radicals resulting from the oxidation of lipids and proteins and DNA fragmentation.

Numerous studies have been conducted on the biological activities of VGSE, for the aforementioned reasons. However, to the best of our knowledge, most of the bioactivities of VGSE demonstrated in the current investigation have not been previously reported. Therefore, the objective of this research was to be the first to report the health-promoting activities of VGSE related to the inhibition of DNA damage, protein damage, and labile iron activity, in addition to inhibiting enzymes, such as Tyrosinase, Porcine α-amylase, and Acetylcholinesterase, whose inhibition has proven positive impact on wide-spread diseases. Additionally, the effect of VGSE on the proliferation of liver, colon, and breast cancer cells was investigated.

## Results and discussion

### Bioactive compounds

#### Total phenolics

The VGSE powder was evaluated for its total phenolic content, which was 14,234 ± 20.34 mg/100 g of VGSE as presented in Table 1. The hydrolysis of the phenolic oligomers and glycosidic monomers present in grape seeds results in the release of distinct phenolic aglycons with further exposed hydroxyl groups that react with the Folin-Ciocalteu reagent^9^. These results are similar to those of previous studies on the polyphenols of grape seed extract, which ranged from 7920 to 44,097 mg/100 g of VGSE^7,9–11^.

**Table 1:** Polyphenolic content of VGSE (mg/100 g). Values are means ± SD of three experiments. Analysis was achieved using Breeze software (version 1.15).

Total phenolics	Total flavonoids	Total tannins	Gallic acid	4-Hydroxy-3-Methoxybenzoic acid	Catechin	Syringic acid	Epicatechin	P- Coumaric acid	Ferulic acid	Rutin	Cinnamic acid
14,234 ± 20.34	6721 ± 14.21	2,143.5 ± 16.45	1.45 ± 0.07	15.21 ± 0.24	58.41 ± 0.56	2.93 ± 0.01	6.40 ± 0.31	49.75 ± 0.26	20.01 ± 0.11	57.26 ± 0.47	6.02 ± 0.14

#### Total flavonoids

Flavonoids constitute a large subclass of phytochemicals that affect metabolic processes and exert positive effects on human health^7,9^. As reported in Table 1, total flavonoids were 6721 ± 14.21 mg/100 g of VGSE. These findings are consistent with those of previous studies^7,9^.

#### Total tannins

The total tannin content was 2143.5 ± 16.45 mg/100 g of VGSE (Table 1). These data are in agreement with those of previous studies^11,12^. The variations in the results for total tannin, phenol, and flavonoid content could be due to various factors. One such factor may be the genetic potential of the individual species for polyphenol biosynthesis. In addition to the genetic (varietal) background, the maturation stage may also be critical^11,12^.

#### HPLC quantification of phenolic acids and flavonoids

Phenolic acid and flavonoid standards were used to identify most of the phenolic compounds in the VGSE. However, other compounds with similar chromatographic behavior and phenolic spectra could not be confirmed because of the unavailability of standard compounds. The phenolic composition of the VGSE is shown in Table 1. The major constituents in VGSE were catechin 58.41 ± 0.56, followed by rutin 57.26 ± 0.47, P- coumaric acid 49.75 ± 0.26, ferulic acid 20.01 ± 0.11, 4-Hydroxy-3-Methoxybenzoic acid 15.21 ± 0.24, epicatechin 6.40 ± 0.31, cinnamic acid 6.02 ± 0.14, syringic acid 2.93 ± 0.01, and gallic acid 1.45 ± 0.07 mg/100 g VGSE. These results were also comparable with those of previous studies on the polyphenols in grape seeds. Some results were in the same range while others were higher or lower than our results. Differences in phenolic compound structures can be influenced by grape variety, geographical and climatic conditions, fertilization, soil, cultivation practices, degree of ripeness, and extraction method^8,13–16^.

### Free radical-scavenging activity

#### DPPH^·^-free radical-scavenging

**Figure 1:**
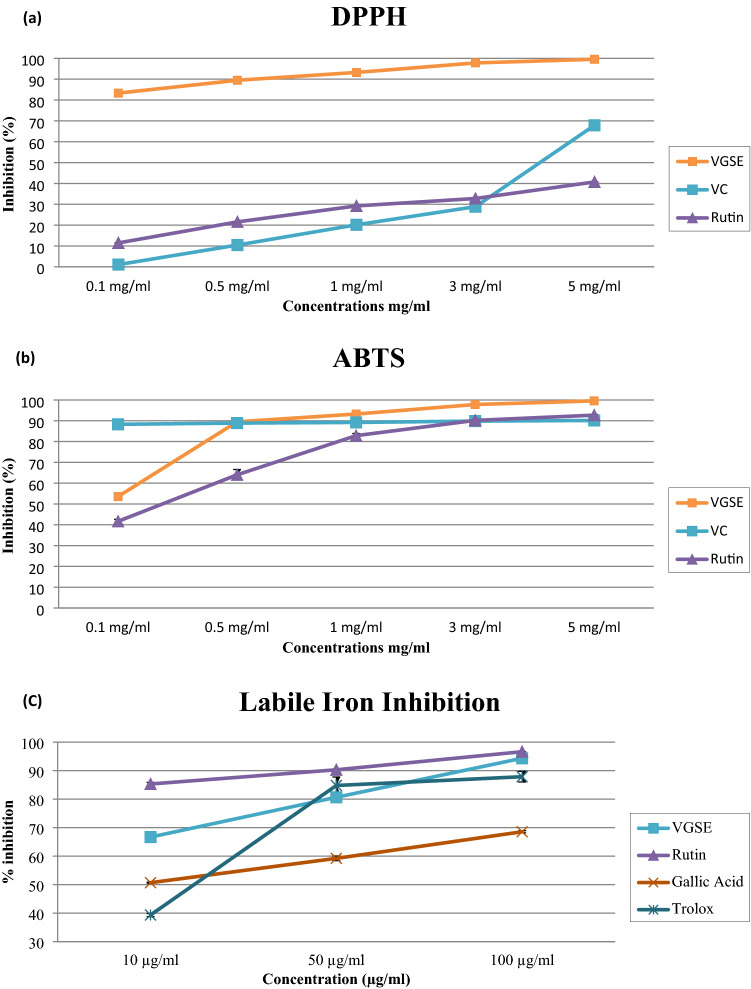
Free radical inhibition activities of VGSE or antioxidant compounds at different concentrations; (**a**) DPPH^·^ inhibition activity, (**b**) ABTS^·^ inhibition activity, and (**c**) Labile iron inhibition activity; Values are means ± SD of three experiments.

DPPH^·^ is a fairly stable free radical that accepts hydrogen radical or electron, thus becoming a stable diamagnetic molecule. It has been widely used to assess the activity of natural antioxidants against free radicals in-vitro^17^. The results of the current investigation indicated that, based on the concentrations used, VGSE significantly (p < 0.05) inhibited DPPH^·^ radical activity as shown in Table 2 for IC_50_ and Fig. 1a. The scavenging activity of DPPH^·^ increased proportionally with extract concentration from 0.1 to 5 mg /mL. Moreover, the results showed that the relative antioxidant activity of VGSE was higher than of those of vitamin C and rutin. The inhibition percentage of DPPH^·^ radicals ranged from 83.33 ± 1.475% to 99.54 ± 0.569% with IC_50_ value of 16.69 ± 0.84 μg/mL, while for vitamin C the DPPH^·^ radical inhibition ranged from 1.09 ± 0.14% to 67.90 ± 0.85% with IC_50_ value of 880 ± 0.52 μg/mL and for rutin, the inhibition ranged from 11.49 ± 0.49% to 40.72 ± 0.90% with IC_50_ value of 415.90 ± 0.54 μg/mL.

**Table 2:** In vitro free radical inhibition activity. IC_50_ (µg/mL) is the concentration of VGSE or antioxidant compounds that can scavenge free radicals by 50%. Values are means ± SD of three experiments. Analysis was achieved using GraphPad Prism software (version 6.0).

DPPH			ABTS			Labile Iron			
VGSE	VC	Rutin	VGSE	VC	Rutin	VGSE	Rutin	Gallic acid	Trolox
16.69 ± 0.84	880 ± 0.52	415.90 ± 0.54	68.11 ± 0.85	0.68 ± 0.12	6.31 ± 1.00	2.74 ± 0.22	0.08 ± 0.14	4.86 ± 0.23	12.57 ± 0.64

#### ABTS^·^- free radical-scavenging

The ABTS^·^ assay was used to assess the ability of the antioxidants to scavenge ABTS^·^ radicals in vitro. ABTS^·^ is produced in the aqueous phase by reacting with a strong oxidizing agent (potassium persulfate) in the presence of ABTS^·^ salt^18^. As shown in Table 2 and Fig. 1b, vitamin C had the highest ability for scavenging ABTS^·^ at the lowest concentration (0.1 mg/mL) with a value of 88.29 ± 0.22% and IC_50_ value of 0.68 ± 0.12 μg/mL, while VGSE and rutin had values of 53.60 ± 1.47% and 48.10 ± 0.07% with IC_50_ values of 68.11 ± 0.85 and 6.31 ± 1.00 μg/mL, respectively. The scavenging capacities of VGSE, rutin and VC increased gradually with increasing their concentration until they reached 99.54 ± 0.59%, 90.13 ± 0.04%, and 92.69 ± 0.32, respectively at 5 mg/mL.

Previous studies have shown that DPPH^·^ and ABTS^·^ scavenging activities correlate well with antioxidant capacity^19^. Significant differences observed among results from different studies can be explained by different phenolic contents, individual species, maturation stage, and seasonal variations^18^.

#### Labile iron inhibitory activity

Iron is essential for many key metabolic processes in the body including DNA synthesis, transcription, and repair, oxygen transport, energy production, drug detoxification, and oxygen storage^20,21^. However, evidence from many studies indicates that the increased generation of superoxide-free radicals in the mitochondria results in the accumulation of cellular iron^22^. Labile unbound iron is highly reactive and potentially toxic because of its involvement in the induction of Reactive Oxygen Species (ROS) formation by the Haber–Weiss and Fenton reactions^20,21,23^. Moreover, labile iron may also lead to ferroptosis, which is a newly discovered form of programmed cell death caused by the iron-dependent accumulation of lipid hydroperoxides^20^.

We previously demonstrated that antioxidants, such as gallic acid, rutin, and Trolox could attenuate the superoxide-dependent release of iron from ferritin in a dose-dependent manner^22^. Therefore, one of the aims of this study was to evaluate whether antioxidant-rich VGSE exert similar effects. The results in Table 2 and Fig. 1c clearly show that VGSE reduced iron release from ferritin in a comparable manner. Rutin was the most effective inhibitor of iron mobilization from ferritin followed by VGSE, gallic acid, and Trolox. The percentage inhibition by VGSE was 66.69 ± 0.30, 80.65 ± 1.13, and 94.35 ± 0.63%, at 10, 50, and 100 μg, respectively with IC_50_ value of 2.74 ± 0.22 μg/mL. Rutin had 85.32 ± 0.56, 90.30 ± 0.01, and 96.65 ± 0.01% inhibition, with IC_50_ value of 0.08 ± 0.14, μg/mL, whereas that for gallic acid was 50.68 ± 0.18, 59.22 ± 0.81, and 68.55 ± 0.38% with IC_50_ value of 4.86 ± 0.23and that for Trolox was 39.33 ± 0.54, 84.79 ± 3.00, and 87.87 ± 1.87% with IC_50_ value of 12.57 ± 0.64, at 10, 50, and 100 μg, respectively. The discrepancies detected among the various antioxidants could be related to their effectiveness in neutralizing superoxide, and the iron-chelating capacity of VGSE and other antioxidants^21,22^.

### Enzyme inhibitory activity

#### Tyrosinase inhibition activity

Tyrosinase plays a crucial role in melanogenesis^24^. The human skin contains melanocytes below the hair follicles and basal epidermis. Melanocytes induce skin pigmentation through melanin production. Generally, melanin synthesis by melanocytes is induced by ultraviolet (UV) light-induced ROS activation by αmelanocyte-stimulating hormone (α-MSH)^25^. Melanin protects the skin by absorbing up to 75% UV radiation and scavenging ROS. However, excessive melanin production may lead to the development of skin diseases such as skin cancer^25,26^.

**Figure 2:**
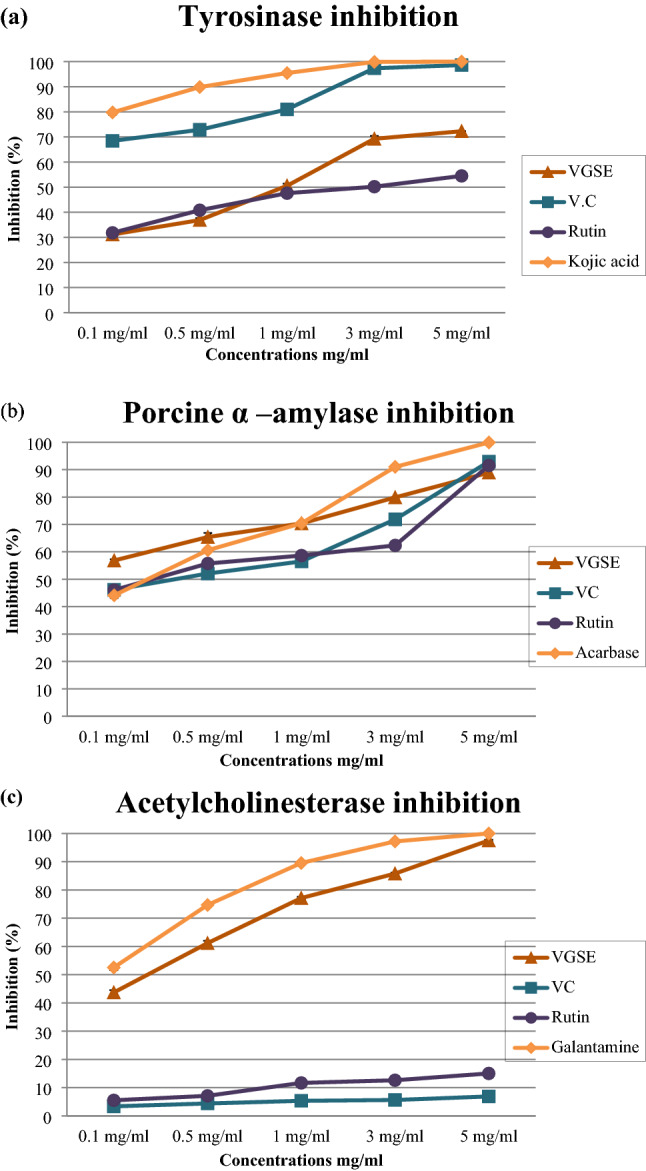
(**a**) Tyrosinase inhibition activity, (**b**) Porcine α-amylase inhibition activity, and (**c**) Acetylcholinesterase inhibition activity for VGSE, antioxidant compounds, or enzyme activity inhibition standards at different concentrations; Values are means ± SD of three experiments.

As shown in Fig. 2a and Table 3, the percentage inhibition of tyrosinase by VGSE ranged from 31.16 ± 1.80 to 72.26 ± 0.06% with IC_50_ value of 501.10 ± 1.00 μg/mL. Tyrosinase inhibition by vitamin C ranged from 68.43 ± 0.22 to 98.62 ± 0.03% with IC_50_ value of 42.74 ± 0.23 μg/mL, inhibition by rutin ranged from 31.80 ± 0.36 to 54.48 ± 1.01% with IC_50_ value of 72.08 ± 0.54 μg/mL, and inhibition by Kojic acid, that is usually used as positive control, ranged from 79.77 ± 0.32 to 100 ± 0.00% with IC_50_ value of 24.25 ± 2.09 at 0.1–5 mg/mL. The results showed that VGSE is a highly potent and selective tyrosinase inhibitor. Nevertheless, Leal. et al.^19^ studies demonstrated that grape stem extracts have anti-tyrosinase activity, with 53.83% inhibition (at 1 mg/mL) of this enzyme activity. This is comparable with our results for VGSE, which showed 50.55% inhibition of this enzyme activity at a concentration of 1 mg/mL. The inhibition reaction might be caused by the hydroxyl groups of the phenolic compounds of the VGSE, which could bind through hydrogen bonding to one or more sites of the enzyme, resulting in lower enzymatic activity. Furthermore, flavonoids are chelating agents due to their polyhydroxy phenolic structure, which can interact with copper ions in the active site of tyrosinase, resulting in inhibition of its activity. Polyphenols and phenolic acids have proven to be suitable inhibitors of this enzyme activity^27^. Yu et al.^28,29^ reported that cinnamic acid and ferulic acid which are included in the VGSE bioactive compound profile were found to effectively inhibit tyrosinase activity and bind to the different sites of tyrosinase, and they were likely to synergistically inhibit the activity of tyrosinase.

**Table 3:** In vitro enzyme inhibition activity. IC_50_ (µg/mL) is the concentration of VGSE, antioxidant compounds, or standard compounds that can inhibit enzyme activity by 50%. Values are means ± SD of three experiments. Analysis was achieved using GraphPad Prism software (version 6.0).

Tyrosinase				Porcine α-amylase				Acetylcholinesterase			
VGSE	VC	Rutin	Kojic acid	VGSE	VC	Rutin	Acarbose	VGSE	VC	Rutin	Galantamine
501.10 ± 1.00	42.74 ± 0.23	72.08 ± 0.54	24.25 ± 2.09	49.47 ± 0.89	87.24 ± 0.71	79.60 ± 0.55	223.6 ± 18.59	165.70 ± 0.55	1113 ± 0.34	421.70 ± 0.60	102.9 ± 9.01

#### Porcine α-amylase inhibition activity

α-Amylase plays a key role in starch digestion and in determining the rate of glucose release. Polyphenols have been suggested to control starch digestion and regulate postprandial hyperglycemia by inhibiting of α-amylase activity^30,31^. The binding affinity of polyphenols to α-amylase has been suggested to play a key role in their inhibitory activity^31^. As shown in Fig. 2b and Table 3, the percentage inhibition of α-amylase by VGSE ranged from 56.84 ± 1.80 to 88.98 ± 1.52% with IC_50_ value of 49.47 ± 0.89 μg/mL. α-Amylase inhibition with vitamin C ranged from 46.13 ± 0.35 to 92.93 ± 0.18% with IC_50_ value of 87.24 ± 0.71 μg/mL, inhibition by rutin ranged from 46.05 ± 0.52 to 91.51 ± 0.51% with IC_50_ value of 79.60 ± 0.55 μg/mL, and inhibition by acarbose, that is usually used as a positive control, ranged from 44.12 ± 0.20 to 99.92 ± 0.12% with IC_50_ value of 223.6 ± 18.59 μg/mL, at 0.1–5 mg/mL. VGSE showed notable inhibitory action against porcine α-amylase which is likely associated with the formation of hydrogen bonds between the binding site of the enzyme and the hydroxyl groups of phenolic acids^32^. Furthermore, phenolic acids have been shown to bind to porcine α-amylase compactly and alter its secondary structure through hydrogen bonding networks, hampering the channel of the substrate into the catalytic site, and leading to the inactivation of porcine α-amylase^32^.

#### Acetylcholinesterase inhibition activity

Acetylcholine (ACh) is an ester of choline and acetic acid discharged at nerve endings as an endogenous chemical that enables neurotransmission. Choline acetyltransferase (AChE) catalyzes the transfer of acetyl groups as substrates from the coenzyme acetyl-CoA into choline producing ACh in specific brain cells, which are known as cholinergic neurons. According to the cholinergic hypothesis, AChE inhibitors lead to an increase in the ACh concentration in the brain and the improvement of cognitive functions in patients with Alzheimer’s Disease (AD)^33,34^. Therefore, the inhibition of acetylcholine esterase can be used as a strategy for the treatment of Alzheimer’s disease. Figure 2c and Table 3 present that the percentage inhibition of acetylcholinesterase by VGSE ranged from 43.70 ± 0.83 to 97.53 ± 0.29% with IC_50_ value of 165.70 ± 0.55 μg/mL. Inhibition of acetylcholinesterase by vitamin C ranged from 3.39 ± 0.28 to 6.89 ± 0.34% with IC_50_ value of 1113 ± 0.34 μg/mL, inhibition by rutin ranged from 5.53 ± 0.63 to 15.03.51 ± 0.86% with IC_50_ value of 421.70 ± 0.60 μg/mL, and inhibition by galantamine, that is usually used as a positive control, ranged from 52.54 ± 0.21 to 100 ± 0.00% with IC_50_ value of 102.9 ± 9.01 μg/mL, at 0.1–5 mg/mL. Our study shows evidence of AChE inhibition activity by VGSE in vitro, which agrees with previous studies^35–37^. The mechanism(s) of inhibition can be due to the interaction of phenolic compounds with the active site of the enzyme as discussed above for tyrosinase and α-amylase^38^.

### DNA damage by free radicals

**Figure 3:**
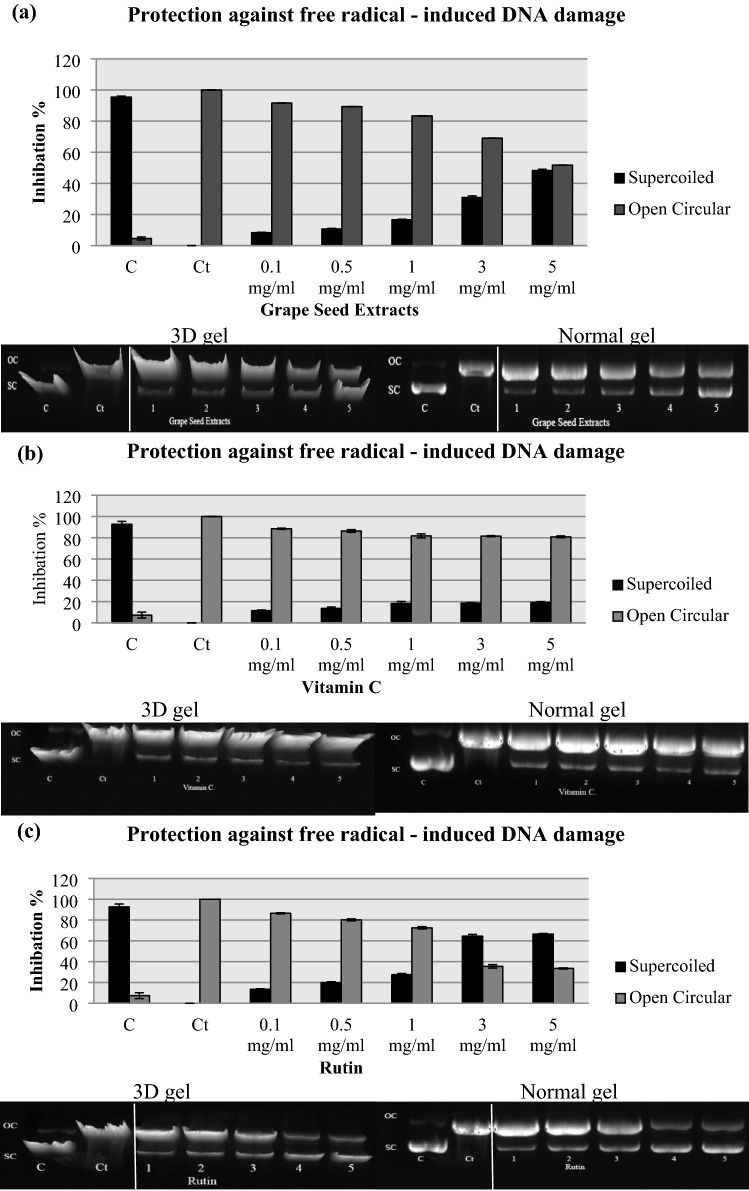
pBR322 DNAdamage inhibition activities of (**a**) VGSE, (**b**) VC, and (**c**) rutin at different concentrations. Lane1’C’: pBR322 DNA; Lane2’Ct’: pBR322 DNA + H_2_O_2_ + UV; Lane 3–7: pBR322 DNA + H_2_O_2_ + UV + VGSE, VC, or rutin at different concentrations (0.1–5 mg/mL); C and Ct are cropped from different parts of the same gel. Values are means ± SD of three experiments. Images were captured using Image lab 4.1 software (version 6.1.0 build 7).

VGSE was examined to determine its protective effect on pBR322 plasmid DNA against the toxic effects of oxidative stress in the presence of UV light and H_2_O_2_, which are highly mutagenic. The results obtained from this experiment are shown in Fig. 3a–c and Table 4. Two plasmid DNA bands appeared during agarose gel electrophoresis; a fast-moving band representing native supercoiled (SC) circular DNA and a slow-moving band corresponding to the open circular (OC) form. The hydroxyl radical (OH^·^) produced from the photolysis of H_2_O_2_ by UV light results in DNA strand scission activity. The hydroxyl radical produced by the reaction between H_2_O_2_ and O2^·−^ causes most of the oxidative damage to biological molecules. Treatment with UV and H_2_O_2_ resulted in complete degradation of plasmid DNA.

**Table 4:** In vitro DNA and BSA damage inhibition activity. IC_50_ (µg/mL) is the concentration of VGSE, VC, or rutin that can inhibit DNA and BSA damage by 50%. Values are means ± SD of three experiments. Analysis was achieved using GraphPad Prism software (version 6.0).

DNA			BSA		
VGSE	VC	Rutin	VGSE	VC	Rutin
14.486 ± 1.05	42.03 ± 1.06	2.45 ± 0.13	15.32 ± 0.43	25.94 ± 0.28	4.42 ± 0.24

The damage was reduced by VGSE, vitamin C, or rutin. The plasmid alone showed 95.41 ± 0.60% SC and 4.59 ± 0.60% OC and 92.70 ± 2.78% SC and 7.30 ± 0.60% OC. On the other hand, the plasmid subjected to H_2_O_2_ and UV showed 100% OC. The electrophoretic pattern revealed that VGSE, vitamin C, and rutin protected the DNA from damage by UV and H_2_O_2._ Pretreatment with VGSE, vitamin C, or rutin reduced DNA strand breaks as evidenced by an increase in the supercoiled form, a subsequent decrease in the open circular form of DNA, indicating recovery of the supercoiled form. The results of VGSE for SC ranged from 8.35 ± 0.24 to 48.28 ± 0.81% with IC_50_ value of 14.486 ± 1.05 μg/mL, whereas those for vitamin C ranged from 11.50 ± 0.63 to 19.10 ± 0.91% with IC_50_ value of 42.03 ± 1.06 μg/mL, and those for rutin ranged from 13.50 ± 0.52 to 66.50 ± 0.57% with IC_50_ value of 2.45 ± 0.13 μg/mL, at 0.1 and 5 mg/mL, respectively. Rutin exerted the greatest protective effect on pBR322 plasmid DNA from damage followed by VGSE and vitamin C. Phenolic extracts from various seeds and plants have been shown by previous research to protect DNA from strand breakage at different concentrations. The present study provides, for the first-time direct evidence of the free radical-scavenging properties of VGSE. The results showed that VGSE, effectively protected plasmid DNA against ionizing radiation, in an in vitro system independent of DNA repair or other cellular defense mechanisms. The ability of VGSE to scavenge (OH^·^) and O2^·−^ may contribute to its protective effects against radiation-induced DNA damage in the pBR322 system.

### Protein oxidation induced by AAPH

**Figure 4:**
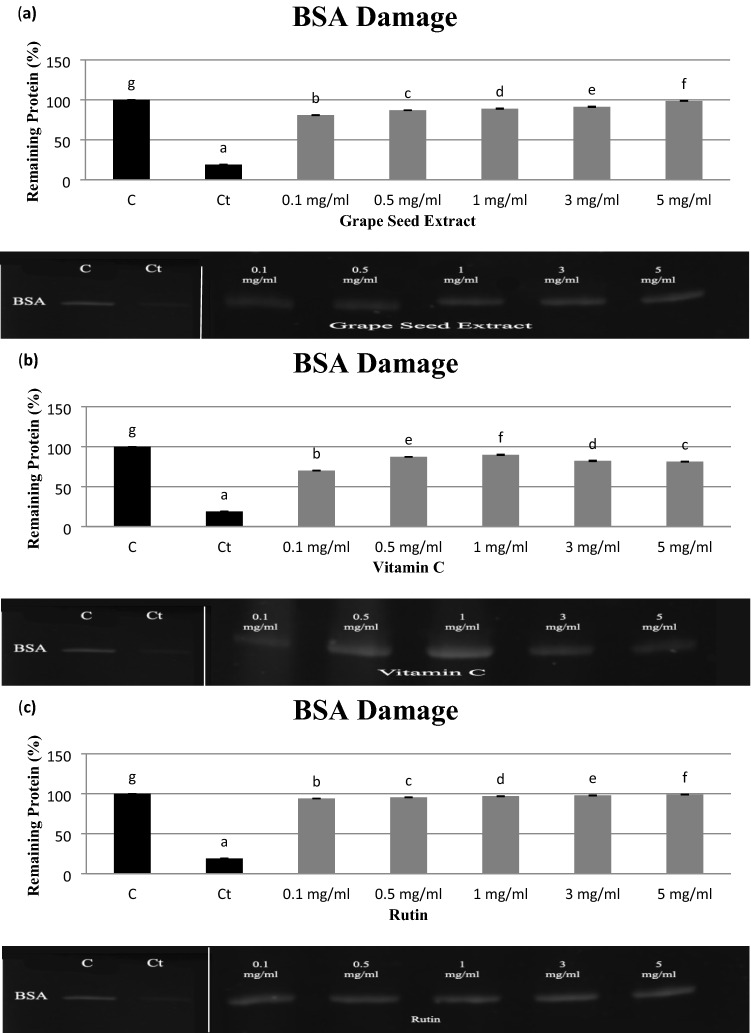
SDS-PAGE of the effects of (**a**) VGSE, (**b**) VC, and (**c**) rutin on the oxidative damage of BSA by AAPH. Lane 1’C’: BSA; Lane 2’Ct’: BSA + AAPH; Lane 3–7: BSA + AAPH + VGSE, VC, or rutin at different concentrations (0.1–5 mg/mL); C and Ct are cropped from a different gel. Values are means ± SD of three experiments. Different letters in the graph denote significant differences, P < 0.05. Images were captured using Image lab 4.1 software (version 6.1.0 build 7).

ROS can damage biological molecules, including proteins, directly and indirectly. Oxidation of proteins by ROS or secondary by-products of oxidative stress results in covalent modification of the protein molecule, which leads to varied functional consequences. Electrophoresis of BSA following incubation for 30 min with APPH in the presence or absence of different concentrations of VGSE, vitamin C, or rutin and the corresponding densitometry analyses of the corresponding bands are presented in Fig. 4a–c and Table 4. The results showed that the band density of control BSA (lane 1) was 100%, while that of treated BSA (lane 2) decreased to 18.98 ± 0.32% after incubation for 30 min with APPH. The treatment with different concentrations (0.1 to 5 mg/mL) of VGSE, vitamin C, or rutin (lanes 3–7) showed a protective effect on BSA damage. Fragmentation of BSA was reduced by 80.92 ± 0.35 to 98.72 ± 0.38% for VGSE with IC_50_ value15.32 ± 0.43 μg/mL, whereas that for vitamin C ranged from 70.13 ± 0.34 to 81.34 ± 0.12% with IC_50_ value 25.94 ± 0.28 μg/mL, and that for rutin ranged from 94.12 ± 0.20 to 99.10 ± 0.24% with IC_50_ value 4.42 ± 0.24 μg/mL at 0.1 and 5 mg/mL, respectively. Only vitamin C showed the (antioxidant-dependent mechanisms) and (prooxidants- dependent mechanisms) at concentrations of 3 mg/mL and 5 mg/mL respectively^7^.

### In vitro cytotoxicity effects of VGSE

The MTT assay is commonly used to evaluate the cytotoxicity of natural compounds and new drugs.

**Figure 5:**
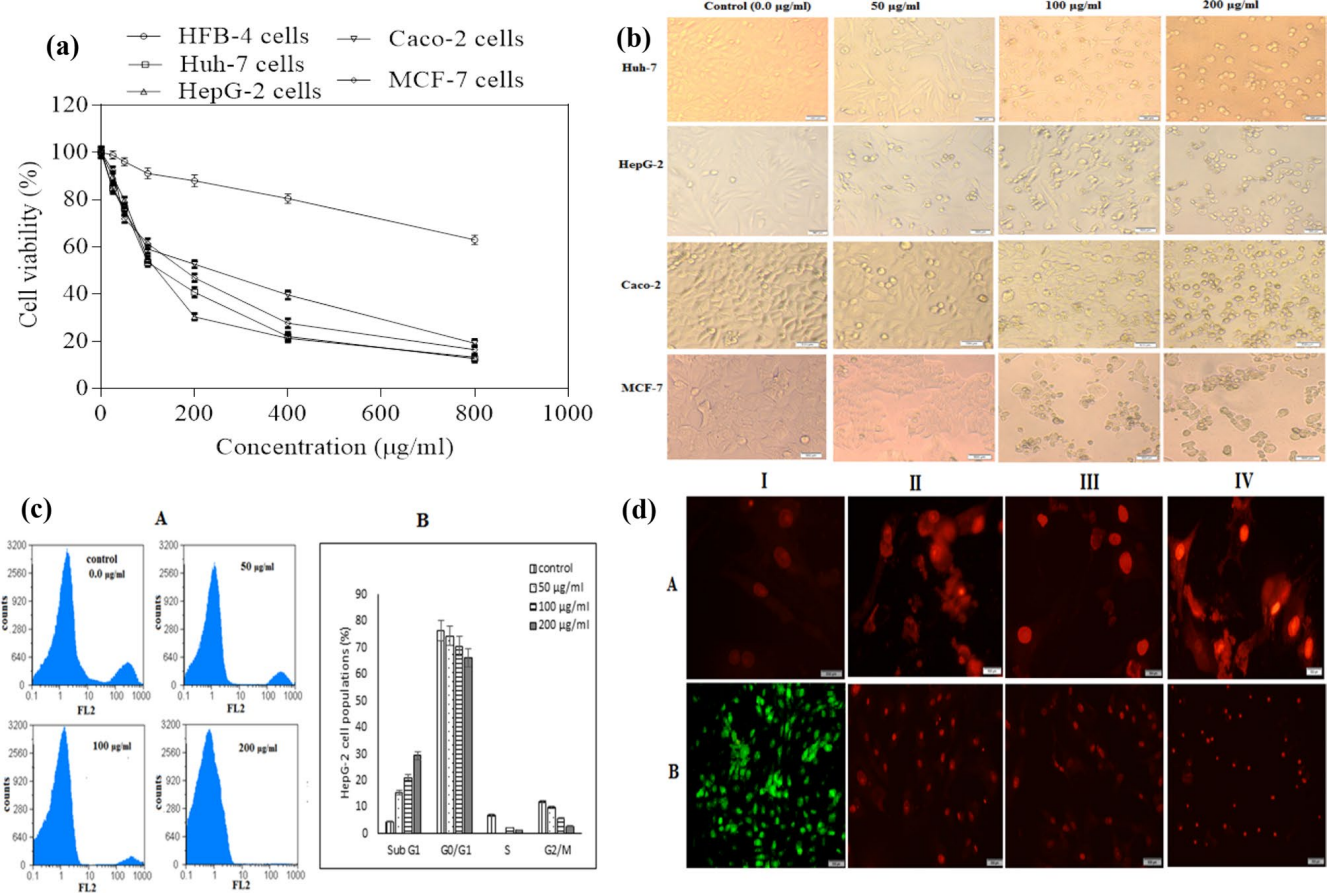
Effects of VGSE treatment at various concentrations for 48 h on (**a**) Cell viability, (**b**) Morphological modifications of cancer cells detected by an inverted phase-contrast microscope, (**c**) Cell cycle analysis in HepG-2 cells: (cA) Original flow charts of cell cycle distribution diagrams and (cB) Quantitative distribution of cell cycle distribution, and (**d**) Apoptosis using PI and double nuclear staining in HepG-2 cells (I, II, III, and IV correspond to concentrations of 0, 50, 100, and 200 μg/ml, respectively): (dA) Fluorescence images of PI staining and (dB) Fluorescence images of ethidium bromide-acridine orange staining. All values are expressed as mean ± SD, n = 3. Images were captured using Cell Quist (version 3.2) and Mod Fit (version 4.0) software.

Hence, the tetrazolium ring is cleaved into MTT formazan crystals by the mitochondrial dehydrogenases found in viable cells. Consequently, the count of viable cells as compared to untreated cells was proportional to the optical density values that resulted from the dissolution of formazan crystals after treatment^39^. Here, we investigated the cytotoxic effects of VGSE on the viability of both normal and cancer cells before and after treatment. The viability of HFB-4 cells (normal human melanocytes) was assessed after treatment with VGSE, and the highest EC_100_ and IC_50_ values indicated the highest safety. The results indicated that EC_100_ and IC_50_ values for VGSE were 16–21 times higher in normal cells than in cancer cells (Table 5). Results also indicate that the grape seed extract had an antitumor effect on Huh-7, HepG-2, Caco-2, and MCF-7 cell lines at IC_50_ values of 134.80 ± 3.04, 125.40 ± 4.89, 156.30 ± 5.39, and 153.80 ± 2.22, respectively, with SI values of 18.98 ± 0.02, 20.41 ± 0.01, 16.37 ± 0.01 and 16.64 ± 0.01, respectively. Furthermore, Fig. 5a shows a highly significant increase in the safety of using VGSE in normal HFB-4 cells with high selectivity against all tested cancer cell lines.

**Table 5:** EC_100_, IC_50_ (μg/mL), and SI values of VGSE against HFB-4, Huh-7, HepG-2, Caco-2, and MCF-7 cell lines after treatment for 48 h. All values were expressed as means ± SD of three experiments. Analysis was achieved using GraphPad Prism software version 6.0).

	HFB-4	Huh-7	HepG-2	Caco-2	MCF-7
EC_100_	25.85 ± 0.15	1.22 ± 0.03	1.27 ± 0.05	1.58 ± 0.05	1.55 ± 0.01
IC_50_	2559 ± 15.12	134.80 ± 3.04	125.40 ± 4.89	156.30 ± 5.39	153.80 ± 2.22
SI	–	18.98 ± 0.02	20.41 ± 0.01	16.37 ± 0.01	16.64 ± 0.01

Figure 5b shows the findings of the proportional morphological analysis of the four cancer cell lines treated with various concentrations of VGSE (50, 100, and 200 μg/mL) for 48 h when compared with untreated cells. All photomicrographs were captured in live-cell mode via inverted microscopy. This showed that the morphologies of all cancer cells under study were changed significantly as a consequence of the treatment. The changed images prove that VGSE causes noticeable selective cell destruction and enhances the modification of cell morphology, which is increased by increasing the dose of treatment in all treated cancer cell lines. These modifications included cell shrinkage, nuclear condensation, and blabbing. Based on these results, it appears that VGSE induces apoptosis to stimulate its antitumor activity. The apoptotic mechanism observed in the present study is in agreement with the findings of Hamza et al. (2018)^7^, which indicate that the anticancer mechanism of grape seed extract is due to the stimulation of apoptosis, decrease in cell proliferation, reduction of oxidative stress, and depression in the regulation of histone deacetylase activity. Apoptosis is a regulated mechanism of cell signalling induced through two main pathways; the extrinsic pathway via activation of death receptors and the intrinsic mitochondrial pathway. Both routes lead to the activation of caspases, the main executioners of cell death^40^. The apoptotic effect of VGSE might be the result of its involvement in numerous cellular signalling alterations^41,42^. Several in vitro and in vivo studies have shown that grape seeds have great anticancer activities against different types of human cancer cells including lung cancer, breast cancer, and gastric adenocarcinoma, however, the agent induces the viability and growth of normal human gastric mucosal cells16 and demonstrates a lack of toxicity in normal cells^43^. In addition, grape seed extract has been shown to exert antiproliferative activity against several types of malignancies including hepatocellular carcinoma^44^, AML^45^, bladder cancer^43^, colon cancer^46^, and pancreatic cancer^47^. Hu et al.^48^ revealed that grape seed extract enhances dose-dependent, mitochondria-related apoptotic effects in human AML cells. Dinicola et al.^49^ revealed that grape seed extract mediates its antitumor activity against both Caco-2 and HCT-8 colon cancer cell lines through the intrinsic apoptotic pathway. Aghbali et al.^50^ revealed that grape seed extract had an antiproliferative effect on oral squamous cell carcinoma via the enhancement of apoptosis with IC_50_ values strongly showing that VGSE had a potent cytotoxic effect on KB cells. These results indicate the selectivity of VGSE on KB cells sparing noncancerous HUVEC cell line, which provides a possibility for its promising applications in the treatment of oral squamous cell carcinoma.

### Anti‑cancer effect of VGSE on cancer cells

#### Cell cycle arrest

Cell cycle arrest is one of the most effective strategies for inhibiting cancer development and growth^51^. The cell cycle in tumor cells is deregulated by the irregular expression of cyclin-dependent kinases (CDK) and cyclin complexes, which are associated with the transition of the G2/M phase^52^. Therefore, targeting abnormal cell cycle progression in cancer cells by deregulating cyclin complexes and CDK expression and phosphatases modulating their function could be significant tools to control the excessive proliferation of cancer cells^53^.

The effect of VGSE on cell cycle arrest in HepG-2 cells was studied to gain insight into the potential cellular mechanisms that induce antitumor effects. HepG-2 cells were treated with VGSE for 48 h at doses of 0 μg/mL (control), 50, 100, and 200 μg/mL. Figure 5c shows the capability of VGSE to enhance the arrest of cell cycle distribution in both main checkpoint phases (G0/G1 and G2/M) of cell population growth. The results demonstrated that the treatment dose-dependently increased the percentage of the apoptotic phase (sub-G1) population, whereas the synthesis (S) phase was diminished by increasing the treatment dose. These results show that VGSE stimulated cell cycle arrest of treated cells in the sub-G1 phase and the S phase as compared with control (untreated) cells, triggering apoptotic mechanisms in a dose-dependent manner. In agreement with our results, recent studies have revealed that grape seed extract arrests the cell cycle in the G2/M phase in HepG-2 cells^51,54^ and colorectal cancer cells^55^. Furthermore, several studies have shown that grape seed extracts induce cell cycle arrest in the G1 phase and modulate key cell cycle-related genes^52,56^.

### Nuclear staining

Advanced confirmation of the potent antitumor activity and apoptotic effect of VGSE was conducted by capturing alterations in the morphology of HepG-2 cells after treatment via nuclear staining with propidium iodide by PI dye and double (EB/AO) staining. As presented in Fig. 5dA, HepG-2 cells seemed to lose their cruciform shape and changed to a round shape in a dose-dependent manner after treatment with VGSE for 48 h has compared with the control (untreated cells). Moreover, staining showed that the nuclei of HepG-2 cells became more intensely fluorescent, and condensed, and showed chromatin fragmentation in a dose-dependent manner after treatment with VGSE. Untreated HepG-2 cells showed insignificant PI-stained cells, and the highest efficacy was observed at the highest concentration of VGSE. Furthermore, EB/AO double nuclear staining revealed the incidence of apoptosis at a late stage in VGSE -treated HepG-2 cells, which lost their cellular integrity and emitted red and orange fluorescence instead of green fluorescence in control (untreated) HepG-2 cells as shown in Fig. 5dB. The detection of DNA fragmentation is considered a promising indicator of late-stage apoptosis. Cancer progression is attributed to the production of reactive oxygen species (ROS), which lead to DNA fragmentation, damage, and mutations as well as chromosomal aberrations^57^ Thus, supplementation of tumor cells with natural candidates having antioxidant properties is required. Among these, grape seed extract has a potent antioxidant property that seems to be greater than those β carotenes, vitamins C and E^58^. Rahimi et al.^59^ revealed that grape seed extract induced the apoptosis of HL‑60 cells in a dose‑dependent manner within concentrations of 25–100 µg/ml, while at a higher concentration than 200 µg/ml, grape seed extract induced cell necrosis. Furthermore, they demonstrated that grape seed extract within a range of 25–100 µg/ml enhanced cell cycle arrest in the G0/G1 phase of HL‑60 cells.

## Conclusion

In conclusion, the results obtained showed that VGSE contains high levels of polyphenols, including phenolic acids, flavonoids, and tannins, and exerts potent antioxidant activity in vitro by effectively scavenging free radicals. These findings also showed that VGSE inhibited DNA damage, protein damage, and labile iron activity. Furthermore, VGSE also inhibits tyrosinase, α-amylase, and acetylcholinesterase. The present study also provides further evidence of the ability of VGSE to induce the death of liver, colon, and breast cancer cells by apoptosis. These findings should further promote the utilization of date seed extract in nutraceutical products and as an ingredient in functional foods and stimulate future research to confirm that VGSE can exert similar effects in vivo and clinical trials.

## Methods

### Plant material and preparation

VGSE powder,100 g (799932307713) was standardized to polyphenol content (95% Proanthocyanidin) and purchased from Nutrients-Scientific Co. *NuSci ® *(Diamond Bar, CA, 91765 USA, www.HerbStoreUSA.com). VGSE was dissolved in a 1:1 methanol/water mixture before application. This solution was then used at a concentration (0.1–5 mg/mL).

### Study materials

Rutin, vitamin C, TPTZ, FeCl3, acetate buffer, ferrous sulfate, 1,1-diphenyl-2-picrylhydrazyl (DPPH^·^), sulfanilic acid, glacial acetic acid, sodium phosphate, naphthyl ethylenediamine dihydrochloride, ammonium molybdate, FeSO_4_, agarose, H_2_O_2_, MTT [3-(4,5-dimethylthiazol-2-yl)- 2,5 diphenyltetrazolium bromide], DMSO, trypsin/EDTA, RNase A, PI dye and ethidium bromide/acridine orange (EB/AO), paraformaldehyde, and pBR322 DNA were purchased from Sigma–Aldrich Chemical Co. (St. Louis, MO, USA). RPMI-1640 and DMEM medium were purchased from (Lonza, USA). Huh-7, HepG-2 (hepatoma), Caco-2 (colon carcinoma), MCF-7 (breast carcinoma), and HFB-4 (normal human melanocytes) cell lines were obtained from ATCC (American Type Culture Collection, Manassas, VA, USA).

### Bioactive components

#### Total phenolics

The total phenolics were assessed by spectrophotometric analysis using Folin–Ciocalteu’s phenol reagent as reported by Habib et al.^60^. The standard curve for total phenolics was compared using a standard solution (0–100 mg mL^−1^) of gallic acid and the total phenolic content was expressed as mg gallic acid equivalent (GAE) per 100 g of VGSE powder.

#### Total flavonoids

Total flavonoid content was estimated using the method reported by Habib et al.^60^. An aliquot (250 μL) of each extract or standard solution was mixed with 75 μL of a 5% NaNO_2_ solution and 1.25 mL of H_2_O. After 6 min, 150 μL of a10% AlCl_3_ solution was added. After 5 min, 1 M NaOH solution (0.5 mL) was added and the total volume was made up to 2.5 mL with H_2_O. The absorbance of the blank was measured at 510 nm wavelength. The results were expressed in terms of mg rutin equivalent (RE) per 100 g of VGSE.

#### Total tannins

Condensed tannins were transformed in presence of concentrated H_2_SO_4_, by the reaction with anthocyanidols to vanillin^11^. 50µL of the seed extract appropriately diluted was mixed with 3 mL of 4% methanol vanillin solution and 1.5 mL of H_2_SO_4_. After 15 min, the absorbance was measured at a wavelength of 500 nm. The total tannin content of VGSE was expressed as mg catechin equivalents (CE) per gram of dry weight using a standard calibration curve with catechin. The range of the calibration curve was 50–600 mg/mL.

#### HPLC quantification of phenolic acids and flavonoids

HPLC-analysis (RP-HPLC) was performed as described by Habib et al.^17^. In brief, with a 1525 Binary HPLC pump (Waters, Milford, MA, USA) separation module equipped with a 717 plus auto-sampler (Waters, Milford, MA, USA), 2487 dual UV. detector (Waters, Milford, MA, USA) operated using Breeze software (version 1.15). The column was Waters Xterra RP 18 5 µm 4.6 × 150 mm. All solvents were HPLC-grade and filtered through a 0.45 µm filter disk. Elution was carried out with 1% acetic acid (solvent A) and acetonitrile (solvent B) with a linear gradient starting with 5% B, to reach 7% at 5 min, 9% at 10 min, 12% at 15 min, 15% at 18 min, 16% at 20 min, 18% at 25 min, 20% at 30 min, 22% at 32 min, 25% at 35 min, 28% at 38 min,30% at 40 min, 31% at 42 min, 32% at 45 min, 34% at 48 min, 35% at 50 min, 40% at 55 min, 50% at 60 min, 95% at 80 min and 5% at 90 min, and post-run for 5 min. All analyses were performed at room temperature, with an injected volume of 20 µL and a flow rate of 0.7 mL/min. The UV spectra were recorded at 280 and 330 nm.

### Free radical-scavenging activity

#### DPPH^·^-free radical-scavenging assay

The free radical-scavenging capacity of rutin, vitamin C and VGSE (0.1–5 mg/mL) was studied by evaluating their free radical-scavenging effect on 1,1-diphenyl-2-picrylhydrazyl (DPPH^·^) radicals. The estimation was based on the method reported by Habib et al.^61^. The results were expressed as the percentage inhibition of the DPPH^·^ radical. The percentage of inhibition of DPPH^·^ radical was calculated using the following equation:

1 $$\% \,{\text{of}}\,{\text{DPPH}}^{ \cdot } \,{\text{inhibition}} = \frac{{{\text{Abs}}\,{\text{control}} - {\text{Abs}}\,{\text{sample}} }}{{{\text{Abs}}\,{\text{control}}}} \times 100$$

where Abs control is the absorbance of DPPH^·^ solution without the tested sample.

#### ABTS^·^-free radical-scavenging assay

The free radical-scavenging capacity of rutin, vitamin C and VGSE (0.1–5 mg/mL) was also studied by evaluating the free radical-scavenging effect on ABTS^·^ radicals. Determination was conducted based on the method reported by Platat et al.^62^. The results were expressed as the percentage of ABTS^·^ radical inhibition calculated using Eq. (1) as described above.

#### Labile iron inhibition assay

The effects of VGSE, gallic acid, rutin, and Trolox on superoxide-dependent iron release from ferritin were evaluated using the methods described previously^22^. Ferritin (100 mg/mL) was mixed with riboflavin (400 mM), methionine (1340 mM), 10, 50, or 100 mg/mL VGSE, gallic acid, rutin, or Trolox. The mixture was illuminated using a fluorescent lamp for 90 min at 25 °C. The labile iron content was measured as described by Ibrahim et al.^22^.

### Enzyme inhibitory activity

#### Tyrosinase inhibition assay

Tyrosinase inhibitory activity was measured as described by Habib et al.^17^. L-tyrosine solution (4 mL) at 0.5 mg/mL was dissolved in 20 mM phosphate buffer at pH 6.8, and then added to rutin, vitamin C, kojic acid and VGSE (0.1–5 mg/mL). After 10 min of incubation at 37 °C, 1 mL of mushroom tyrosinase (50 units/mL, dissolved in 0.2 M phosphate buffer, pH 6.8), was added to the mixture. The absorbance was recorded after 10 min at 475 nm. A 50% ethanol solution was used as a blank, and 1 mL of deionized water was used as the control. % Tyrosinase inhibitory activity was calculated according to *Eq. (**1**)* as described above.

#### Porcine α-amylase inhibition assay

Inhibition of porcine α-amylase activity was described by Habib et al.^17^. A mixture of 50 µL of rutin, vitamin C, acarbose and VGSE (0.1–5 mg/mL), and 50 µL of 0.02 M sodium phosphate buffer at pH 6.9, with 6 mM sodium chloride including α-amylase solution, 13 units /mL. For 10 min, the mixture was incubated at 25 °C, and then 50 µL 1% starch solution in 20 mM sodium phosphate buffer at pH 6.9 with 6 mM NaCl was added. The mixture was incubated at 25 °C, followed by addition of a color reagent (1 mL dinitrosalicylic acid). To stop the reaction, the mixture was kept for 10 min in a water bath at 100 °C and then cooled to 25 °C. 1 mL of deionized water was added, and the absorbance was read at 540 nm using a 96-well microplate reader. Inhibition of porcine α-amylase was estimated using *Eq. (**1**)* as described above.

#### Acetylcholinesterase inhibition assay

Anti-AChE inhibition activity was determined as described by Habib et al.^17^. 325 µL buffer Tris–HCl 0.05 M, at pH 8, plus 100 µL of rutin, vitamin C, galantamine and VGSE (0.1–5 mg/mL), with 25 µL anti-acetylcholinesterase, 0.28 U/mL, were incubated for 15 min. Consequently, 75 µL of 15 mM acetylcholine iodide (AChI) solution, and 475 µL. DTNB solution (3 mM) was added. The final mixture was incubated at 25 °C for 30 min, and at 405 nm the mixture absorbance was measured. The AChE inhibition was estimated using Eq. *(**1**)*, as described above.

### DNA damage by free radicals

The assay was performed using the method described by Habib et al.^17^. Briefly, 6 μL of 30% H_2_O_2_ was added to an Eppendorf tube with a volume of 20 μL containing 6 μL of PBS buffer, 0.2 μg of pBR322 DNA in 2 μL of 50 mM PBS (pH 7.4), and 4 μL of samples. The reactions were initiated by UV irradiation and sustained for 5 min on the surface of a UV transilluminator TFM-26 (UVP, Upland, CA, USA), with an intensity of 25 W cm^−2^ at 312 nm at room temperature. At the end of the reaction, the samples were run on 0.8% agarose. The gel was stained with ethidium bromide and photographed and analyzed using Image lab 4.1 software (version 6.1.0 build 7). Vitamin C and Rutin were used as positive controls.

### Protein oxidation induced by AAPH

Alkylperoxyl radical-induced oxidation of Bovine Serum Albumin (BSA) was performed as described by Habib et al.^17^. In brief, BSA (0.5 mg mL^−1^) was incubated with AAPH (20 mM) in the presence or absence of different extracts in a shaking water bath at 37 °C for 30 min. A protein sample without AAPH was used as control. After treatment, protein samples were mixed with loading buffer and heated at 100 °C for 5 min. An aliquot of each sample was loaded and separated using 10% SDS-PAGE under reducing conditions. The free stained gels were imaged using a ChemiDoc MV gel documentation system (Bio-Rad, Hercules, CA, USA). To determine the amount of protein damage, band intensity was determined using the Image Lab 4.1 software (version 6.1.0 build 7) (Bio-Rad, Hercules, CA, USA). The optical density of each band was determined and standardized for the control group.

### Cytotoxicity of VGSE on HFB-4 cells

To evaluate the cytotoxicity of VGSE at various concentrations on normal cells, the hydrogen acceptor method of MTT [3-(4,5-dimethylthiazol-2-yl)- 2,5 diphenyltetrazolium bromide] was used, which is considered a highly accurate and rapid colorimetric method as described by^63^. The HFB-4 (normal human melanocytes) cell line was seeded into a sterile 96-well microplate and cultured in DMEM (Lonza, USA) supplemented with 10% fetal bovine serum (FBS) at 5.0 × 10^3^ cells per well. After incubation for 24 h, various concentrations of VGSE at 0.0, 25, 50, 100, 200, 400 and 800 μg/mL were added to the cells in triplicate. After another incubation for 48 h in a 5% CO2 incubator, the cells were washed 3 times for removing debris and dead cells, then 200 μL of MTT (Sigma-Aldrich) at a concentration of 0.5 mg/mL was added to each well, and the cells were incubated at 37 °C for 2–5 h. After removing the MTT solution, 200 μL DMSO was added, and the absorbance was measured at 570 nm using a microplate reader (BMG LabTech, Germany). The safe dose (EC_100_) and half-maximal inhibitory concentration (IC_50_) values of VGSE were analyzed using the GraphPad Prism software (version 6.0).

### Anti‑cancer effect of VGSE on cancer cells

#### MTT assay

The anticancer effect of VGSE was tested in vitro using the Huh-7 and HepG-2 (hepatoma), Caco-2 (colon carcinoma), and MCF-7 (breast carcinoma) cell lines. Huh-7 and Caco-2 cells were maintained in DMEM supplemented with 10 FBS, whereas HepG-2 and MCF-7 cells were maintained in RPMI-1640 (Lonza, USA) supplemented with 5% FBS. All cancer cells (5.0 × 10^3^ cells/well) were seeded in sterile 96-well tissue culture microplates and incubated for 24 h for attachment. Various concentrations (0.0, 25, 50, 100, 200, 400, and 800 µg/mL) of VGSE were added to the cells in triplicate, and cells were incubated in 5% CO2 at 37 °C for 48 h. The cytotoxic effect of VGSE on the tested cancer cells was evaluated using the MTT assay as described above. IC_50_ and EC_100_ values of the VGSE were analyzed using GraphPad Prism software (version 6.0) and the selectivity index (SI) values were reported as the ratio of the IC_50_ on normal human cells (HFB-4) versus the IC_50_ value of each tumor cell line^39,64^. In addition, the effect of VGSE at concentrations of 50, 100, and 200 μg/mL on the morphology of all tested tumor cells was studied by phase-contrast microscopy (Olympus, Germany) and compared with a negative control (untreated cells).

#### Cell cycle analysis

The cell cycle of treated HepG-2 cells was investigated by flow cytometry in comparison with untreated cells^39^. After treatment of HepG-2 cells with VGSE at concentrations of 0, 50, 100, and 200 µg/mL, approximately 1.0 × 10^6^ cells were de-attached using trypsin/EDTA and resuspended in 500 μL of 1.0 × PBS, pH, 7.2. After that, the cells were collected by centrifugation at 1200 rpm for 5 min at 4 °C and fixed by adding a dropwise 700 μL of 70% cold ethanol during a gentle vortex. HepG-2 cells were washed three times with cold 1.0 × PBS, pH 7.2, and incubated in 500 μL of PBS containing 5 μg/mL RNase A (Sigma-Aldrich) for 1 h at 37 °C. Then 10 μL of PI (Sigma-Aldrich) was added to cells at a final concentration of 1 mg/mL in deionized water and incubated at 4 °C until analysis in the dark condition. The cell cycle of HepG-2 cells before and after treatment was analyzed by FACS (Partec, Germany) using Cell Quist (version 3.2) and Mod Fit (version 4.0) software at 488 nm.

#### Nuclear staining analysis

The anti-cancer effect of VGSE on HepG-2 cells was investigated by one and two fluorescent nuclear staining methods using PI dye and ethidium bromide/acridine orange (EB/AO) dye. HepG-2 cells were cultured in sterile 24 well plate and treated with different concentrations of VGSE as mentioned above. After washing the cells three times with cold PBS, they were fixed with 4% paraformaldehyde and stained with PI (10 µg/mL) or EB/AO (100 µg/mL for each dye) for 20 min. The cells were washed with cold PBS and cell images were visualized and captured using a fluorescence phase-contrast microscope (Olympus, Japan) with a dichromatic mirror cut-at 505 nm and an excitation filter (480/30 nm). Untreated cells were used as the negative controls.

#### Statistical analysis

All analytical determinations were performed in triplicates. Statistical Analyses were performed using SPSS for Windows (version 20; SPSS Inc., Chicago, IL, USA). The results obtained were analyzed using analysis of variances to estimate the significance (P < 0.05) of the main effects. Values of different parameters are expressed as the mean ± standard deviation.

## Supplementary Information

Supplementary Information.

## Data Availability

The authors declare that all data supporting the findings of this study are available within the paper and its associated files.
