# Supplementary material for: Grape seed proanthocyanidin extract inhibits DNA and protein damage and labile iron, enzyme, and cancer cell activities
Source: Sci Rep. 2022 Jul 20;12:12393. doi: 10.1038/s41598-022-16608-2 (PMC9300616; doi:10.1038/s41598-022-16608-2)
Supplement: Supplementary file 1 — Supplementary Information. [file 41598_2022_16608_MOESM1_ESM.docx]

***Supplementary Information***

**Grape seed proanthocyanidin extract inhibits DNA and protein damage and labile iron, enzyme, and cancer cell activities**

**Hosam M. Habib*^1^, Esmail M. El Fakharany^2^, E. Kheadr^1^, and Wissam H. Ibrahim^*3^**

^1^Functional Foods and Nutraceuticals Laboratory (FFNL), Dairy Science and Technology Department, Faculty of Agriculture, Alexandria University, Alexandria, Egypt.

^2^ Protein Research Department, Genetic Engineering and Biotechnology Research Institute GEBRI, City for Scientific Research and Technology Applications, New Borg EL Arab 21934, Alexandria

^3^ Department of Nutrition and Health, College of Medicine and Health Sciences, United Arab Emirates University, Al Ain PO Box 15551, UAE.

***Correspondence**

Dr. Hosam M. Habib, ^1^Functional Foods and Nutraceuticals Laboratory (FFNL), Dairy Science and Technology Department, Faculty of Agriculture, Alexandria University, Alexandria, Egypt. Contact No: +201003991550, e-mail: [hosamL@hotmail.com](mailto:hosaml@hotmail.com)

Dr. Wissam H. Ibrahim, Department of Nutrition and Health, College of Medicine and Health Sciences, United Arab Emirates University, Al Ain PO Box 15551, UAE. Contact No: +971507131826, e-mail address: [wibrahim@uaeu.ac.ae](mailto:wibrahim@uaeu.ac.ae)

***Supplementary Figure 3***


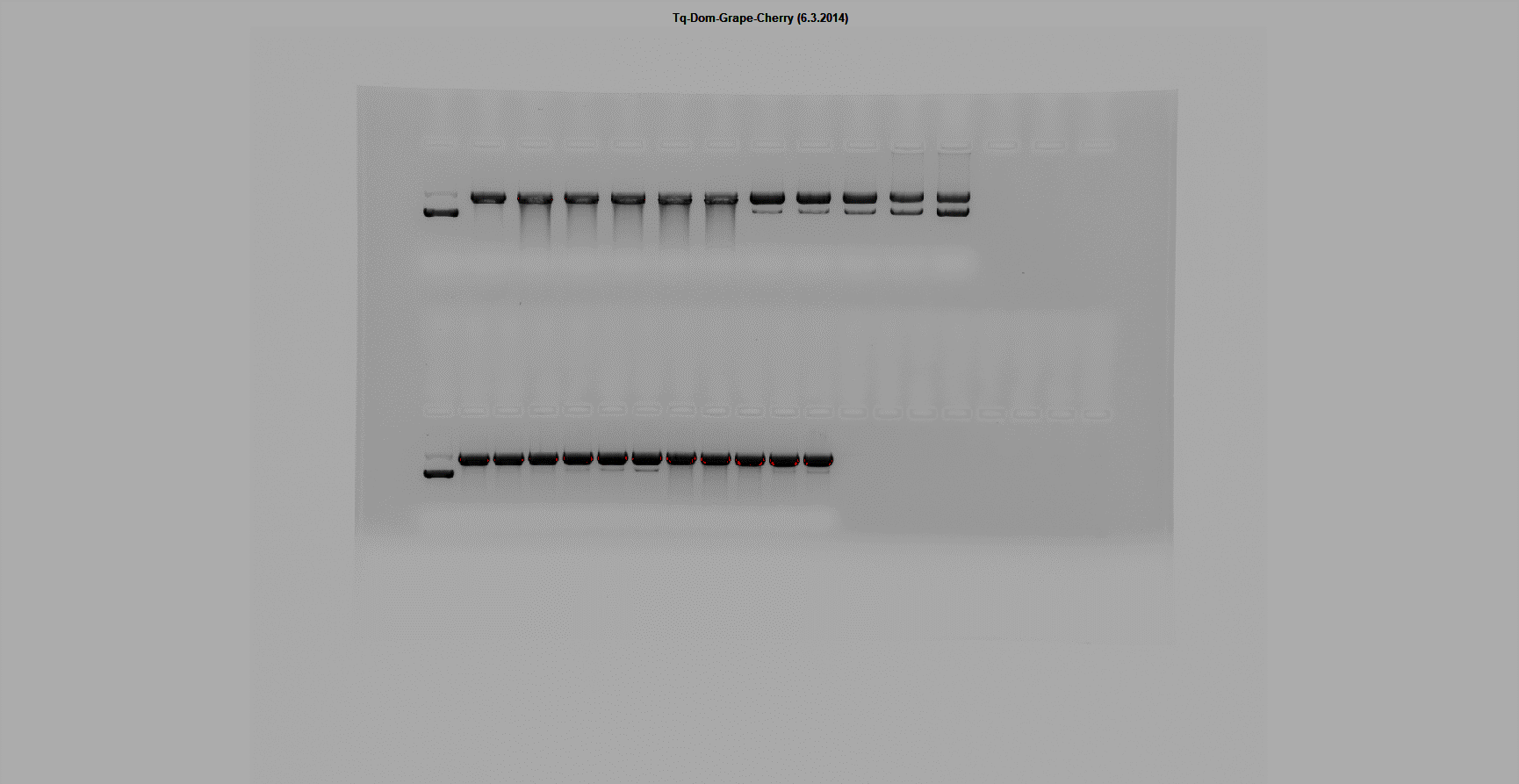


VGSE

1 2 3 4 5

C Ct

**
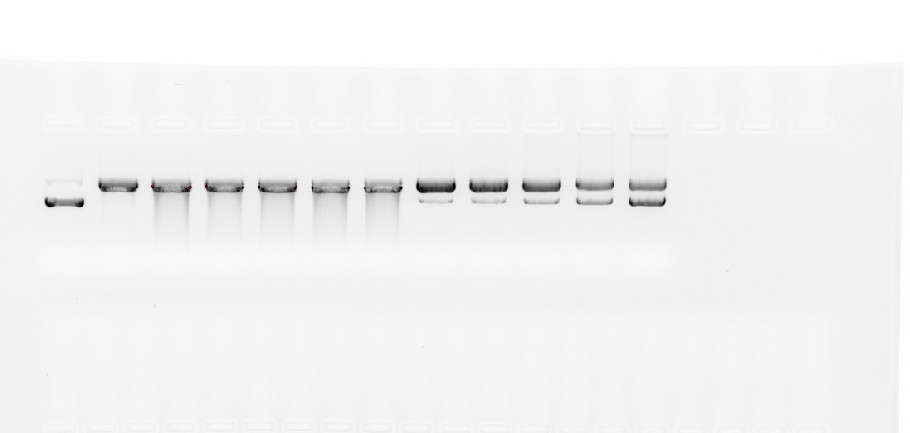
Supplementary Figure 3. The original gel of figure 3a**


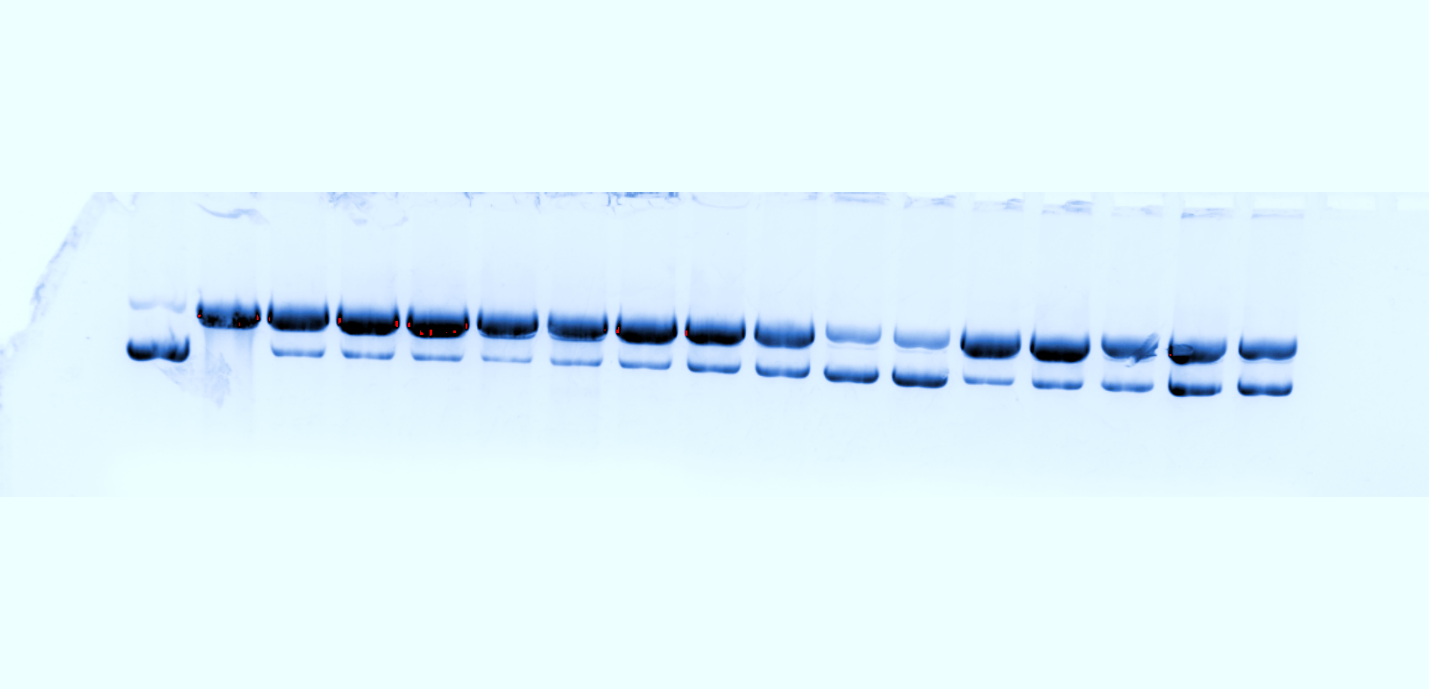


VC Rutin

1 2 3 4 5 1 2 3 4 5

C Ct

**Supplementary Figure 3. The original gel of figure 3b&c**

***Supplementary Figure 4***


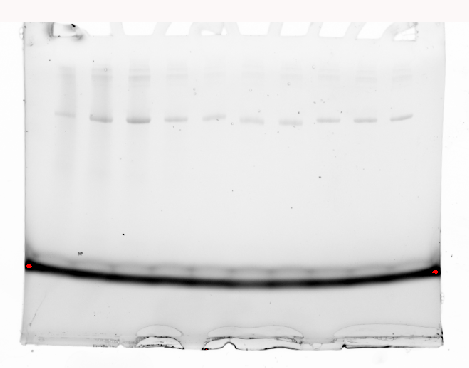


VC VGSE

1 2 3 4 5 1 2 3 4 5


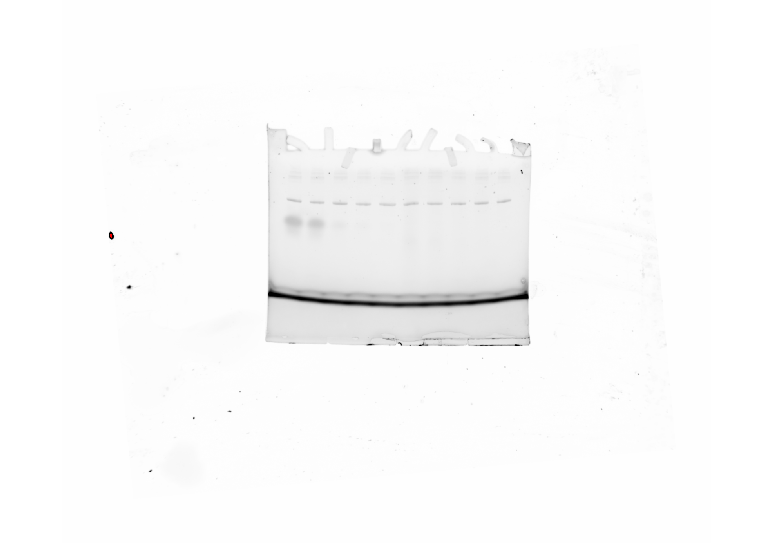
**Supplementary Figure 4. The original gel of figure 4b&a**

Rutin

1 2 3 4 5

**Supplementary Figure 4. The original blots of figure 4b&a**

**Supplementary Figure 4. The original gel of figure 4C**


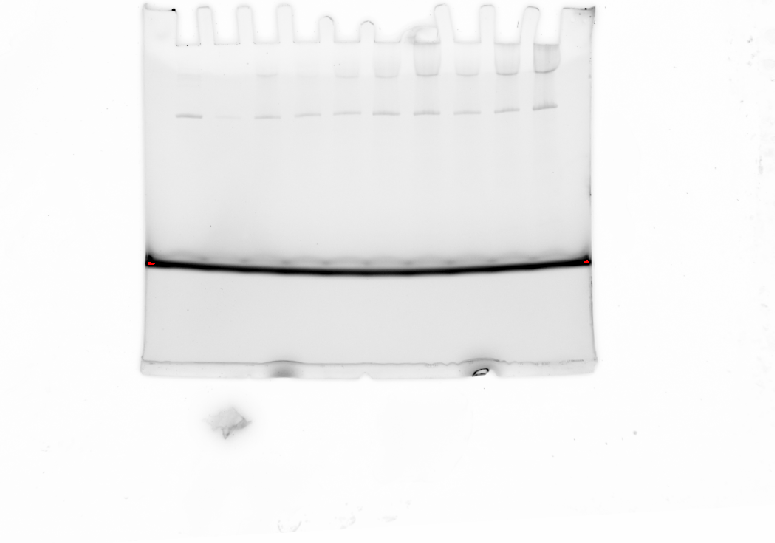


C Ct

**Supplementary Figure 4. The original gel of control and Ct for figure 4a, b &c**
